# Feasibility, Psychosocial Effects, Influence, and Perception of Elastic Band Resistance Balance Training in Older Adults

**DOI:** 10.3390/ijerph191710907

**Published:** 2022-09-01

**Authors:** Nichola M. Davis, Andy Pringle, Anthony D. Kay, Anthony J. Blazevich, Danielle Teskey, Mark A. Faghy, Minas A. Mina

**Affiliations:** 1 Department of Sport, Outdoor and Exercise Science, School of Human Sciences & Human Sciences Research Centre, University of Derby, Kedleston Road, Derby DE22 1GB, UK; 2 Centre for Physical Activity & Life Sciences, University of Northampton, Northampton NN2 7AL, UK; 3 Centre for Human Performance, School of Medical & Health Sciences, Edith Cowan University, Joondalup, WA 6027, Australia

**Keywords:** postural control, elderly, resistance bands, falls prevention, physical activity

## Abstract

This study utilised feedback from older adults during balance-challenging, elastic band resistance exercises to design a physical activity (PA) intervention. Methods: Twenty-three active participants, aged 51–81 years, volunteered to perform a mini balance evaluation test and falls efficacy scale, and completed a daily living questionnaire. Following a 10 min warm-up, participants performed eight pre-selected exercises (1 × set, 8–12 repetitions) using elastic bands placed over the hip or chest regions in a randomised, counterbalanced order with 15 min seated rests between interventions. Heart rate (HR) and rate of perceived exertion (RPE) were measured throughout. Participant interview responses were used to qualify the experiences and opinions of the interventions including likes, dislikes, comfort, and exercise difficulty. Results: Similar significant (*p* < 0.01) increases in HR (pre- = 83–85 bpm, mid- = 85–88 bpm, post-intervention = 88–89 bpm; 5–6%) and RPE (pre- = 8–9, mid- = 10, post-intervention = 10–11) were detected during the PA interventions (hip and chest regions). Interview data revealed that participants thought the PA interventions challenged balance, that the exercises would be beneficial for balance, and that the exercises were suitable for themselves and others. Participants reported a positive experience when using the PA interventions with an elastic band placed at the hip or chest and would perform the exercises again, preferably in a group, and that individual preference and comfort would determine the placement of the elastic band at either the hip or chest. Conclusion: These positive outcomes confirm the feasibility of a resistance band balance program and will inform intervention design and delivery in future studies.

## 1. Introduction

Fall-related accidents are a key national UK public health priority with fragility fractures estimated an annual cost of GBP 4.4 billion [1]. Furthermore, the impact of fall-related accidents may lead to injury, pain, fear of falling, loss of independence and confidence, mobility limitations, and mortality [2]. Age-related declines in both the sensory (vision, vestibular, and proprioception) and neuromuscular (strength, power, range of motion) systems negatively affect postural stability, which is an important factor for older adults to perform usual activities of daily life such as walking, turning, moving, and functioning independently [3]. Impaired balance control increases the risk of falls in older adults due to the difficulty in controlling balance recovery reactions, e.g., swaying around the ankles or hips, and taking steps beyond the base of support [4,5]. Therefore, developing physical activity (PA) interventions to improve/maintain postural control is important not only for overall health and psychological benefits but also for daily functioning and independence [6]. Understanding the key facilitators and barriers that older adults may face when adopting and maintaining PA is crucial when developing feasible, accessible, appropriate, affordable, and enjoyable interventions [7], including those centred on fall prevention.

The determinants for participation in PA by older adults have been identified in the development of the social ecological model (SEM) [7], which highlights enjoyment, sociable, affordable, accessible, and flexible as important aspects of a PA program for older adults [7]. Additionally, the self-determination theory of human motivation supports the concept of psychological needs, autonomy competence, and enjoyment as influences on behavioural motivation for participation in PA [8,9]. These findings emphasise the importance of obtaining feedback on newly designed PA interventions to ensure that they meet the needs of older adults. Many PA interventions examine task-specific reactive balance, which is recommended as an optimal intervention for improving reactive balance in older adults [5,10]. Perturbation-based balance training aims to incorporate repeated postural perturbations to evoke instantaneous rapid balance reactions to reduce falls in older adults [11,12,13] with the potential of producing quicker adaptations to improve balance compared to conventional balance training [13]. Furthermore, perturbation-based balance training is an approach used in fall prevention with improved balance, confidence [14], resilience, and balance reactive control to respond to real-life circumstances such as trips that occur in daily life [15]. Such interventions are often performed in clinical settings and are not always accessible to all older adults as part of a regular exercise regime.

Muscular strength is another important factor for maintaining balance, which is recommended in PA guidelines for older adults [16]. Strength reductions in ageing have been associated with muscle changes, leading to increased risk of falls and difficulty in performing daily tasks such as climbing stairs, rising from a chair, and household chores. The importance of muscular strength highlights the need to include strengthening exercises in PA interventions for older adults, which can be achieved using elastic band resistance [17]. Studies documenting the use of elastic band resistance versus conventional resistance training using weight machines have shown similar improvements in strength [18], isometric force [19], peripheral muscle force [17], functional exercise capacity [20], and improvements in health-related quality of life in older adults [19]. Elastic band resistance is a useful, cost-effective, and safe intervention in the rehabilitation of balance impairments in older adults [21]. When used in a full-body training program, elastic band resistance has been shown to improve postural control in older adults [22,23], and in targeted lower limb strengthening training has been shown to influence balance, gait function, flexibility, and fall efficacy [24]. However, the importance of involving and engaging older adults in the development of interventions that meet their needs and preferences is a critical factor for a successful PA intervention [7,25]. Furthermore, to our knowledge, the use of elastic band resistance to challenge balance using this methodology within the individual’s base of support (similar to that of perturbation-based balance training) has not been previously examined. Given the above, this study aimed to develop a novel PA intervention using elastic band resistance training to challenge balance and utilise the perspectives of older adults to shape an accessible, appropriate, and acceptable intervention for older adults to meet their needs.

## 2. Materials and Methods

### 2.1. Participants

Twenty-three healthy and moderately physically active older adults (66.5 ± 8.3 years) (Table 1) were recruited through existing physical activity networks in the Derbyshire community to participate in the study after completing written, informed consent and a PA health screening questionnaire. Inclusion criteria included individuals (1) over 50 years of age, (2) having the ability to walk without a walking aid, (3) achieving an activity level score of >600 MET-min per week according to the International PA Readiness Questionnaire (IPAQ), and (4) achieving a score of >23 in the Mini-Mental State Examination (MMSE). Exclusion criteria included participants with (1) unstable cardiovascular conditions, (2) acute respiratory disease, (3) Parkinson’s disease, (4) Huntington’s disease, (5) acute stroke, (6) lower limb paresis, or (7) uncontrolled diabetes mellitus. Fall history was recorded for the previous 6 months with no participants reporting a fall. A falls efficacy scale, International Activity of Daily Living Questionnaire, and a mini balance evaluation test were completed prior to taking part in the PA intervention. This study was conducted as part of a feasibility trial designed to evaluate the intervention design of a novel PA intervention using elastic band resistance. Attendance of a single experimental trial at the University of Derby (UK) was required. A mixed-methods design was implemented using qualitative and quantitative data to help evaluate and understand the feasibility of the PA interventions. Ethical approval was granted by the Human Science Research Ethics Committee at the University of Derby (ID: ETH2021/4503) in July 2021. The study was clinically registered (ClinicalTrials.gov ID: NCT04932408).

### 2.2. Study Design

**Figure 1:**
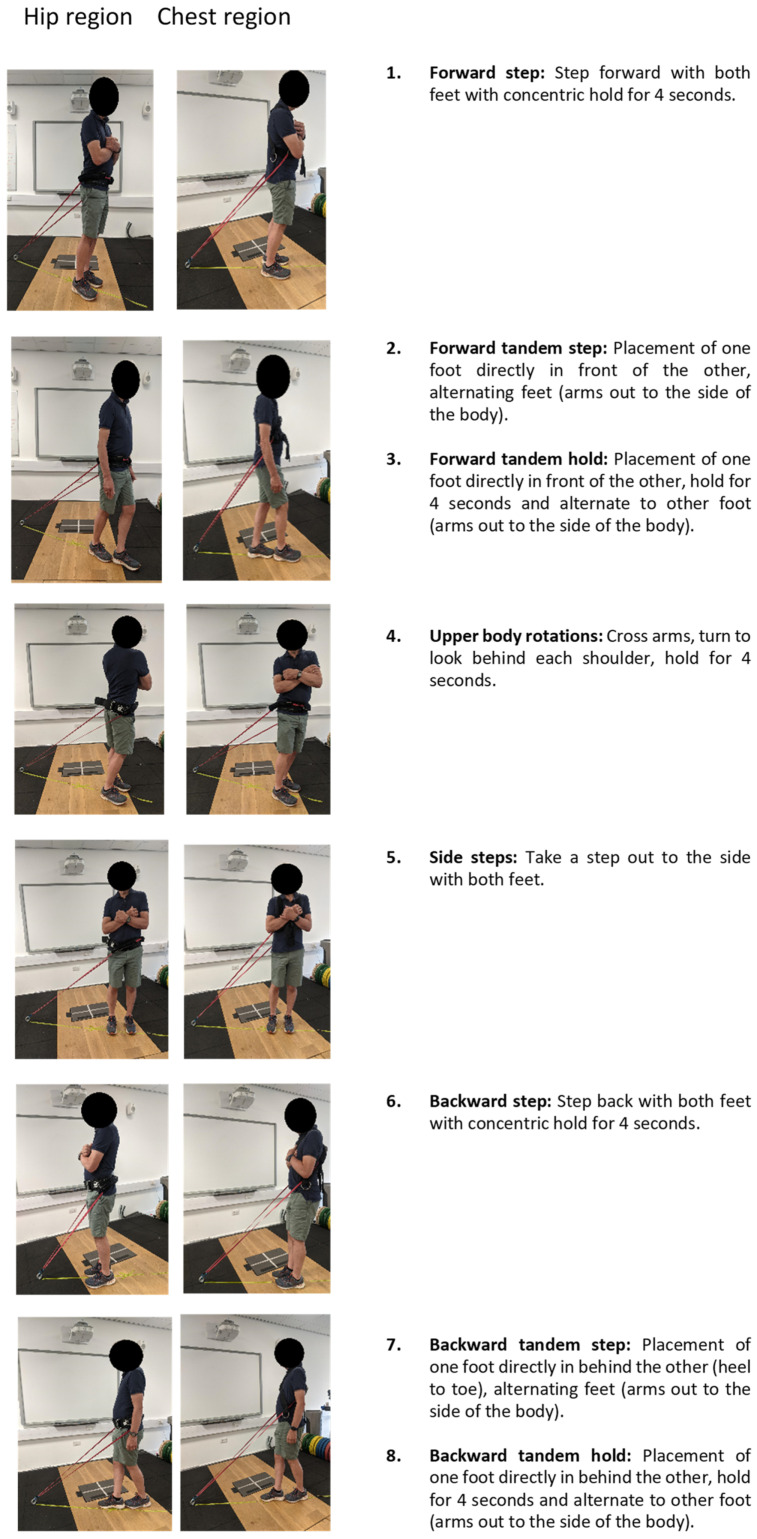
Examples of the elastic band resistance exercises in each PA intervention (hip and chest regions).

**Figure 2:**
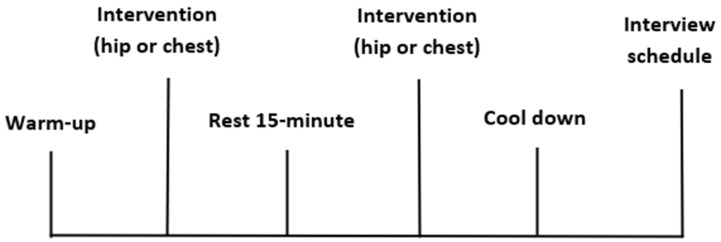
Study design timeline of the intervention protocol.

A randomised, counterbalanced design was implemented to compare two PA interventions using elastic bands placed at the hip and the chest region on the same day in a single session. Each participant performed the intervention between 10:00 a.m. and 2:00 p.m. to control for time-of-day effects on balance performance, including accumulation of general tiredness and general muscle fatigue. Randomisation was performed for both the order of the PA interventions (either the hip or chest placement first) and the order in which the exercises were performed. Following a 10 min supervised warm-up, all participants performed 8 pre-selected exercises for 1 × 12 repetitions (performed for 30 min on average) (Figure 1) with the elastic band placed at either the hip or chest with a 15 min seated rest before completing the second intervention with the elastic band placed at either the hip or chest (Figure 1 and Figure 2). Heart rate (HR) and rate of perceived exertion (RPE) were recorded before, during (6th repetition), and after each exercise. Refinement of the methods during the piloting phases of the study can be found in Supplementary File S1. Following the PA interventions, participants were invited to take part in a semi-structured interview with a digital voice recorder and written notes taken during the interview.

### 2.3. Interviews with Participants

Interviews took place between July and October 2021 with data collected using semi-structured interviews lasting approximately 30 min. The aim of the interviews was to identify the determinants, opinions, and perceptions of the PA intervention to shape subsequent intervention design. This included scaling questions on difficulty, comfort, and enjoyment of the exercise selection and further questions related to personal preferences on the exercise selection, comfort, difficulty, modality, suitability, perceived benefits, safety, environmental factors, and the exercise equipment to determine the feasibility of the PA intervention. The interview schedule was developed through prior knowledge of creating PA interventions [7], and this type of approach has been used previously in PA research to provide in-depth and insightful accounts with professionals [26] and older adult participants [27,28]. The interview schedule is available upon request from the corresponding author.

### 2.4. Data Analysis

Analyses were performed using SPSS (version 26, IBM Corporation, Armonk, NY, USA) with all data reported as mean (M) ± standard deviation (SD). Normal distribution was assessed using the Shapiro–Wilk test; no significant difference (*p* > 0.05) was detected in any variable, indicating that all data sets were normally distributed. Separate two-way repeated measures ANOVAs (2 × 3) were used to determine between (condition × 2) and within (time × 3) effects on HR and RPE measures. Post hoc t-tests were performed to determine the location of any significant differences.

Participant interviews were recorded and manually transcribed verbatim by the lead researcher; all names were altered to a pseudonym code for use in the results to ensure anonymity. Following reading of the transcripts to saturation, template analysis was performed, which encourages the use of initial themes. The priori themes included the exercise band placement, environmental factors, and the exercise preferences. This type of analysis uses a hierarchical coding and offers researchers a high degree of structure; however, this approach also allows for flexibility of the structure, which was required for the analysis in this study [29,30]. Template analysis was used to guide a thematic analysis process [31]: (1) coding to identify interesting features of the data; (2) grouping into sub-themes to provide a systematic framework, and (3) a visual map developed by hand to show the themes and their relationships. Three main themes were defined: (1) perceptions on exercise selection, (2) opinions on the exercise equipment/elastic band placement location, and (3) participant views on the environment factors. These finalised themes were used to guide the analysis and organise the qualitative findings.

## 3. Results

### 3.1. Demographics and Participant Profile

Table 1 and Table 2 show the demographics of the interviewed participants.

### 3.2. Interviews with Participants

**Figure 3:**
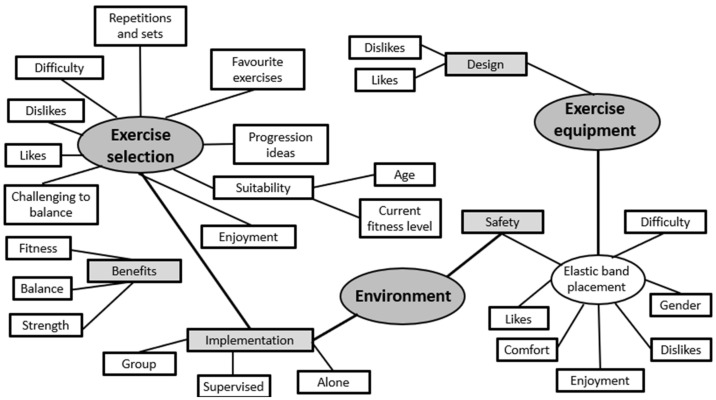
Figure to illustrate the key themes and the interconnectivity of the themes and sub-themes.

Participant reports were organised using themes and sub-themes (see details below). Following analysis, priori themes were merged into three final themes based on the emerging data set: (1) perceptions of the exercise selection, (2) opinions of the exercise equipment, and (3) insights into the preferred environment (Figure 3). Multiple data sets describe the key findings of the participant feedback on the PA intervention through the interview schedules performed and the perceived intensity of the PA intervention recorded by HR and RPE measures.

### 3.3. Participants’ Perceptions of the Exercise Selection

#### 3.3.1. The PA Intervention and Balance

Participants reported their perceptions on the exercises that challenged balance whilst performing them using an elastic resistance band. This is illustrated by the following quotes:

- “The tandem steps, it seemed to me that you only have to be a fraction out and the bands sort of accentuate it and pull you off balance further”. (P19)
- “I just felt a bit out of sync, you know, wobbly again. That’s when I’m putting my foot back like that (tandem stance) I find them a bit wobbly. It does challenge your balance, but it’s nice. It’s all right”. (P14)
- “Definitely everything involving going backwards. Especially if you need to do it under pressure, you know because you’re doing it with band you are you are doing it against something rather than just doing it without as just walking does”. (P4)

Participants reported some of the exercises did not challenge their balance as such:

- “Upper body rotations did not challenge my balance, not at all. And the side steps not at all. I seem to function well stepping sideways”. (P9)
- “I wouldn’t have said the walking one challenged the balance too much (forward and backward steps). But I thought that was quite a good exercise because it felt like to me like walking uphill and it’s got to be got to be good for you. That might be the most cardio-intensive one you know”. (P19)

Overall, the PA intervention was perceived to challenge the balance and stability of most participants.

#### 3.3.2. Comfort of the Exercise Selection

Participants reported their opinions on the comfort of performing the exercises using the elastic resistance band at the hip and chest. This is demonstrated on a level of scaling and by quotes below (Table 3 and Table 4):

- “It was the effect of the toe to heel and holding that position (tandem steps and tandem holds). It was according to question my ability to hold that position heel to toe. My balance didn’t seem to be adequate. I felt less balanced doing them”. (P1)
- “I think going backwards if I’m honest, it’s a cognitive thing, thinking about going backwards with resistance band type harness on feels slightly less comfortable”. (P15).

Furthermore, holding the arms out to the sides during the exercises was an element of the exercises that participants felt was uncomfortable:

- “The only slight issue was that my arms got slightly tired after being held out for as long as it took; but not really an issue”. (P18)
- “When you’ve got the arms out there, we did a number of repetitive ones (exercises) and the upper arms here were starting to ache. So, whether you can reduce that by spreading it around a bit so you’re not you’re not doing repetitive ones of that. It’s not, you know, it’s not painful”. (P23)

The comfort of the exercises in the intervention was also associated with a perception of enjoyment, the ability to perform the exercises, and a sense of security, which was reported by participants:

- “Yeah, they were all comfortable actually. I enjoyed it a lot really”. (P12)
- “It’s not physically uncomfortable, none of them were physically uncomfortable. It just felt a lot a lot more secure while doing it” (exercises in the forward direction) (P19)

#### 3.3.3. Preference for the Exercise Selection

Personal preferences for the exercises that were selected were reported by participants on a level of scaling and quotes. (Table 5 and Table 6):

The participants reported their preference for the exercise selection:

- Forward step: “Personal favourite, I think. But I found that it’s not too hard, so that’s the reason why I’m saying it isn’t it, but I think that these ones the tandem ones, balancing ones I like them as well because I feel that it’s challenging you. So, I have to say that I think that they are probably the most beneficial out of all of them. I quite like that one backwards (backward tandem steps) that challenges you backwards”. (P4)
- “I like the side steps because I felt it on my sides, and I thought that is really good. I did like this (tandem holds) because I was pushing and then I felt it in my arms as well. I liked the twist (upper body rotations)—for me the release of my back muscles really”. (P11)

Several participants reported what they disliked about the exercises:

- “The only ones I didn’t like was the placement of the feet one in front of the other (tandem holds) and having to hold. It’s the balance aspect”. (P1)
- “The parallel steps or the little fairy steps (tandem steps) or whatever they are called I definitely didn’t, but again, it may well be doing you very good”. (P19)
- “Probably one I disliked was the sides the side steps only on the left-hand side. I had to step back with my left foot. I found out my left side is my weaker side anyway, so it’s even proved it even more now so”. (P21)

Performing exercises backwards was highlighted as the least favourite exercise of participants:

- “I’m okay with the backward ones. It’s just that you can’t see behind you. It can be a bit disconcerting. I wouldn’t say I dislike them, but you know, if I didn’t have to do it then I wouldn’t bother doing it”. (P3)
- “Backward steps, they were difficult again for not seeing. And then the tandem one I couldn’t do that at all anyway”. (P6)

#### 3.3.4. Difficulty of the Exercise Selection

Participants reported their opinions on the difficulty of performing the exercises using the elastic resistance band around the hip and chest. This is demonstrated on a level of scaling and by the quotes below (Table 7 and Table 8):

- “I found them [the exercises] difficult, challenging to do not the end of the world thing but just felt a little bit challenging. It’s just the balance I guess, which I know is the object to this in a lot of ways. But as I say, they’re harder (tandem steps), but maybe in terms of this whole thing they become very important”. (P19)
- Tandem steps and tandem holds: “I think anything to do with like the balance is harder, but it’s supposed to be right? It’s challenging more than just stepping forward, it is challenging new balance, isn’t it?”. (P4)

In other cases, participants did not perceive the exercises to be difficult to perform:

- “There aren’t too tough and definitely weren’t too easy, and I wouldn’t even say they were too tough”. (P2)
- “I don’t think any of them seemed really difficult. I would say it was quite easy to do them once I got my head in mind with what I’m supposed to be doing. I didn’t find them difficult, any of them difficult”. (P15)

#### 3.3.5. Heart Rate and RPE Measures

**Figure 4:**
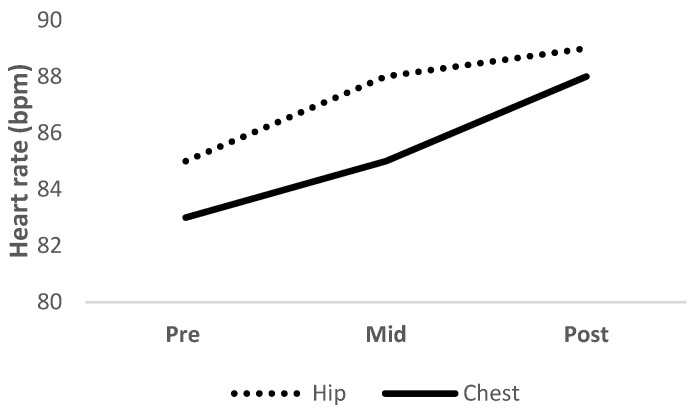
Heart rates measured at pre-, mid-, and post-intervention with the elastic band placed at the hip (dotted line) and chest (solid line).

The two-way mixed model ANOVA revealed a significant main effect of time (*F* = 60.888, *p* < 0.001, *η*_*p*_^2^ = 0.74) but not condition (*F* = 3.567, *p* = 0.07, *η*_*p*_^2^ = 0.14) for HR. Post hoc within-subject analyses revealed significant (*p* < 0.01) increases in HR from pre- to mid- to post-intervention in hip (pre- = 85 ± 11 bpm, mid- = 88 ± 11 bpm, post-intervention 89 = ± 11 bpm, 5%) and chest (pre- = 83 ± 12 bpm, mid- = 85 ± 12 bpm, post-intervention = 88 ± 12 bpm, 6%) conditions (Figure 4).

**Figure 5:**
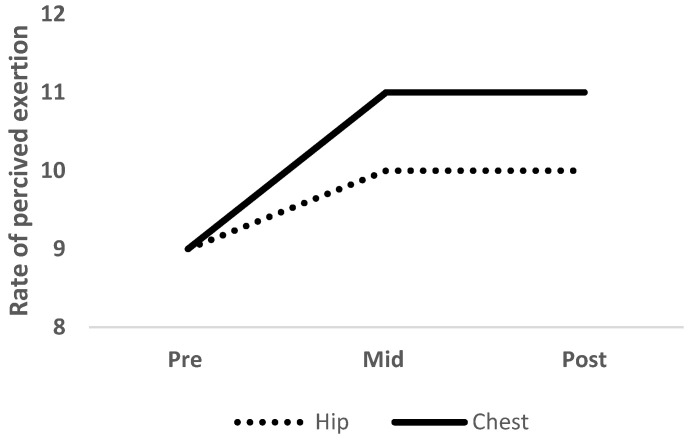
Rate of perceived exertion measured at pre-, mid-, and post-intervention with the elastic band placed at the hip (dotted line) and chest (solid line).

The two-way mixed model ANOVA revealed a significant main effect of time (*F* = 23.433, *p* < 0.001, *η*_*p*_^2^ = 0.52) but not condition (*F* = 3.477, *p* = 0.08, *η*_*p*_^2^ = 0.14) for RPE. Post hoc within-subject analyses revealed significant (*p* < 0.05) increases in RPE from pre- to mid- to post-intervention in hip (pre- = 8 ± 2, mid- = 10 ± 2, post-intervention = 10 ± 2) and chest (pre- = 9 ± 2, mid- = 10 ± 2, post-intervention = 11 ± 2) conditions (Figure 5).

#### 3.3.6. Organisation of the Exercise Selection

Participants reported on the way that the exercises were organised. The repetitions and sets of exercises were perceived to be adequate. Participant responses were as follows:

- “I thought that was adequate. No, I don’t think you could delete any of those and I can’t think of anything that you could add”. (P1)
- “I think that was ample. Yeah, that was ample to do that was just a right level from my perspective as a person, it was right for me”. (P3)

However, in some cases, participants felt that the repetitions and sets of exercises could have been increased.

- “I think for me I could have done more repetitions, um, but again that would have to be assessed depending on each individual”. (P11)
- “It was just starting to make me get into that zone. For others, I can imagine that being too much and for others again who are at the fitter end of the spectrum wouldn’t have found the earlier ones (exercises) particularly demanding”. (P20)

#### 3.3.7. Suitability of the PA Intervention

Participants discussed their personal views on how the PA intervention was suitable for themselves and other people within their age group:

- “I would have thought so, yes. I mean, everybody is individual”. (P8)
- “Yes, it’s a good idea because balance is our main problem as we get older”. (P14)

In some cases, participants felt the exercises were not age-appropriate:

- “No, I don’t. I don’t know really because I don’t find them particularly difficult, but I can imagine somebody who’s not used to it, because I do other things. I wouldn’t know if anybody else would find them difficult. Yeah, that all depends on the level of ability really doesn’t it really”. (P9)
- “For me, not particularly, but I can imagine if I give it 10 years, well, hopefully 20 years they will be”. (P18)

#### 3.3.8. Perceived benefits of the PA intervention

Participants discussed why they felt the exercises were beneficial, as illustrated in the following cases:

- “I just think they give a good workout on the hips and the legs and the knees. I can see the benefits of that, you know”. (P3)
- “They might very well be doing you a lot of good, particularly the side (side steps). I don’t know what it is, but there is something about that I just feel I ought to do that more. I don’t know, just something about that exercise that just feels as though it’s doing me good, without being painful. It feels like something that you ought to include in regular exercise”. (P19)

#### 3.3.9. The Psychosocial Impact of the PA Intervention

Participants discussed the psychosocial impacts of the PA intervention. In particular, participants discussed their level of confidence in performing the exercises:

- “It’s confidence, it’s your confidence knowing you can do it” (P13)
- “I think it’s a confidence thing with where you’re putting your feet as well. How far I felt I could put my feet forward or backward depending on which exercise I was doing and how hard I was pushing against the bands”. (P15)

Participants reported if anxiousness was an aspect that they experienced when performing the exercises (Supplementary File S2).

Participants reported if the exercises made them focus their attention on the task at hand:

- “I thought the others were good because they were challenging and you had to stay focused, particularly number two and three (tandem steps and tandem hold) you had stay focused”. (P11)
- “I was probably flagging a little bit in terms of concentration, not in terms of physical energy, but I think you know after a bit when you’ve been doing things that you have to really think about what you’re doing a bit harder”. (P15)

All the participants reported a positive experience regarding their participation and that they would perform the exercises again (Supplementary File S2).

### 3.4. Opinions of the Exercise Equipment

#### 3.4.1. Preferences for the Elastic Band Placement

Participants reported what they specifically liked about performing the exercises with the elastic band placed at the hip:

- “I could feel the tug when It was on the hips, you know, so I knew I was working extra hard with it on the hips”. (P3)
- “I think it’s the right tension” (P9)

The participants reported their opinions on the safety aspect and what they liked about performing the exercises with the elastic band placed at the chest:

- “I think it’s the right tension” (P9)
- “I thought the harness work very well indeed. In all of them. I just felt better with it like that than I did in the hip position. I did prefer the harness a lot but particularly when you were facing away from the anchor”. (P19)

Furthermore, participants discussed what they disliked about performing the exercises with the elastic band placed at the hip:

- “My thing is just about the band not having the stability on the first lot of exercises, whereas with the harness [chest region] you’ve got more of the stability because I think it detracts from your concentration of what you’re doing”. (P3)
- “You don’t seem to have support with the hip one. It can slip at any time. I’ve got love handles so it sits on me but for somebody who hasn’t got enough I imagine it’s very hard”. (P6)

Participants also stated what they disliked about the chest:

- “I think it was just uncomfortable. It wasn’t the exercises, it was just the feeling of. It being uncomfortable”. (P4)
- “It was a bit restricted on the chest. It wasn’t so enjoyable as with the bottom one”. (P6)

In some cases, female participants reported how they felt restricted wearing the chest harness equipment with the elastic band placed at the chest (Supplementary File S2).

#### 3.4.2. Difficulty of Exercises Performed with the Elastic Band Placed at the Hip

The difficulty of performing the exercises was further discussed in terms of the elastic band placement:

Hip Region (Figure 1).

- “It was difficult at the hip. You’ve got more support with the chest one”. (P6) “With it round your hips I found more challenging. I felt as if I was going to get pinged back”. (P7)
- “It was more challenging to use center of balance and more around your center [hip region] than on top where you can lean into it, if you know what I mean. You can’t use your body weight on it. It’s more attacking your balance from your hips”. (P21)

Chest Region (Figure 1).

- “Chest, I just felt there’s more resistance”. (P18)
- “With your upper half you’ve got the whole of your body, whereas with your lower half you’re using your bottom part, so I’ve found it easier with the whole of my body [chest region]. If you’re pushing something, you push it with the whole of your body so it’s easier, isn’t it?”. (P11)

#### 3.4.3. Preferred Elastic Band Placement

Participants’ preference for their preferred elastic band location while performing the exercises was reported. This is demonstrated in the table and quotes below (Table 9).

- “It just depends on the equipment whether there is a different form, a different way that you could do the hip one. It would probably be the hip one if there was a change”. (P6)
- “I don’t have a problem providing somebody can come up with a solution involved in the band around the waist (hip region). I could feel it slipping so that it that was a bit of an issue I suppose”. (P22)

The safety element in terms of the exercise equipment was an aspect reported by participants with the elastic band placed at the chest (Table 6), which they felt provided a greater sense of safety.

- “Chest, only that the Velcro seemed to hold better there than at the hip”. (P1)
- “Safer, definitely with the chest harness on”. (P5)
- “I don’t think there was any difference for me. I think that I felt okay at either”. (P11)

### 3.5. Environment

Supervision, clear verbal instruction on how to perform the exercises, and a safety briefing on the exercise equipment were factors reported to help to create a sense of a safe environment (Supplementary File S2).

#### 3.5.1. Safety Factors

- “Just realising the band was holding and having you three around me”. (P8)
- “I knew what to expect because you had explained to me about the bands, so I was fully informed, you created a safe environment”. (P2)
- “I know you were there. I trusted you that it’s hooked into the ground, so that’s fine. It’s not going to come out”. (P12)

#### 3.5.2. Preferred Setting

Performing the exercises in a supervised/group setting was preferred by participants.

- “I’d prefer a group setting. Fellow participants would encourage each other. There’d be the social aspect because there’d be an interchange of conversations, whereas. If you’re on your own there wouldn’t”. (P1)
- “My preference would be with other individuals or certainly with an instructor”. (P23)

#### 3.5.3. Shaping the PA Intervention

Participants provided their own ideas about how the intervention design could be shaped, including aspects that could be done differently.

Equipment:

- “I think the band would be better hugging the hip a bit. It could mould itself more into the hip than that then it won’t be sliding down the leg”. (P3)
- “If you had a belt that you could strap round to whatever size you want your body was and had two little clips on the side which fit to the band”. (P6)
- “I think if it were a little bit wider (the elastic band) it might be a little bit more comfortable”. (P9)
- “I would suggest doing it at the side of the hips so that you’ve got an anchor point for front and rear, so they somehow have a pully up or anything like that they are going to feel uncomfortable because it’s actually pulling on part of the body”. (P21)
- “Another belt with it like one that does the weightlifting sticks of Velcro on that using one of them”. (P22)
- “Whether you could adapt a system around your waist which will actually take this the slippage away, that would help”. (P23)

Adaptations:

- “Incorporate more things like a weight”. (P5)
- “What might have been good is if you’ve got like your line which you have over there (marker on the ground) then you’ve got different markers. So, you’ve got a progression in your mind”. (P11)
- “I think the other thing is to apply this to different activities or something like that will also help”. (P12)
- “I guess you could even do like a circuit, you know where you go from each one in turn and then go through them again”. (P19)
- “So, it incorporates it so it’s a bit more of a program almost. Little lunges that’s sort of thing. What we do we always try to do a warmup then a workout and then a warm down”. (P20)
- “Whether you could think about introducing a stronger band, I don’t know. When you’re doing the same band, it’s going to get to get monotonous because I’m going to find it easier and easier and easier”. (P22)

## 4. Discussion

In the current study, the feasibility of a novel PA intervention using elastic band resistance at the hip and chest to challenge balance in older adults was evaluated. The study was designed to include multiple data sets to inform the design of the PA intervention by using interviews to investigate their personal preferences, difficulty, comfort, suitability, perceived benefits, safety, exercise equipment, and the environment. Additionally, quantitative data of HR and RPE were used to determine the PA intensity. These multiple data sets were combined with the feedback from older adults as a unique aspect to the study, which contributes to informing the process of designing PA interventions that are feasible for older adults [1,32,33]. The benefits of involving older adults in the development of such interventions has been shown to encourage older adults to be physically active [34].

The present study identified the feasibility of a novel elastic band resistance PA intervention and the importance of delivery to make older adults feel comfortable and safe, including a sense of enjoyment and considering the environment as well as the preferences and physical abilities of each individual. Therefore, it is important to discuss the outcomes of the study, which include considerations that may be useful for future PA interventions to meet the needs of older adults.

### 4.1. Perceptions of the Exercises

Participants varied in their perceptions of how challenging the PA intervention was regarding balance. However, exercises including the upper-body rotations and the side steps were perceived to be less challenging. These exercises were performed with a wider base of support, which is a likely explanation for the greater stability experienced, influencing participants’ perception that they insufficiently challenged balance [35].

In terms of difficulty, participants suggested that the chest exercises were easier to perform as they could use the force of their whole body to lean against the resistance band to maintain balance, whereas hip exercises were restricted to using the force of the lower body only and were perceived to be more difficult. Furthermore, this may be due to activation of additional muscles, including those in the trunk/core area, which may provide greater benefit to balance performance compared to hip exercises [36].

Holding the arms out to each side of the body during the tandem exercises (Figure 1 (Images 2,3,7,8)) was perceived to increase exercise difficulty. This may be due to the additional cognitive demand of performing two tasks simultaneously, requiring greater coordination [36]. Previous studies have highlighted an increase in perceived difficulty in dual-task exercises and a decrease in balance performance [37,38,39]. Providing the option of a dual-task element in the PA intervention would therefore provide an additional challenge to increase difficulty for people who require it, allowing greater individualisation of the training prescription and potentially improving intervention effectiveness.

#### 4.1.1. Perceptions of Age and Balance Capacity

According to the verbal feedback, participants who perceived themselves to be older also tended to view their balance as poorer (Section 3.3.4, Section 3.3.6). As ageing is associated with a reduction in physical function, including reduced muscle strength, coordination, and balance [40], this is likely explained by age-related fear of falling [41,42].

#### 4.1.2. Psychological and Motivational Considerations

The participants’ perception of their ability to perform the exercises was related to confidence, self-efficacy, and comfort (Section 3.3.9), which are determinants of PA for older adults [43]. Participants expressed that they felt less comfortable performing the exercises that most challenged balance (i.e., tandem steps, tandem holds, and performed exercises backwards ), and this likely placed increased demand on postural control and coordination due to the narrow base of support. Most participants did not report anxiousness whilst performing the exercises, possibly due to the close supervision by staff, with whom the participants were well-acquainted [44]. Furthermore, other factors that likely contributed to participants’ confidence levels were the clear verbal instruction for how to perform the exercises and a detailed safety briefing on the PA equipment to provide everyone with the confidence to perform the exercises (Section 3.5.1). Previous research has highlighted benefits of providing both verbal and written instructions to increase compliance and motivation regarding PA in younger adults [44,45], which provides a sense of security and safety during the intervention. The current intervention considered this aspect within the design with older adults and highlighted the importance of this aspect when creating PA interventions for this age group.

#### 4.1.3. Perceptions of the Elastic Band Placement

Participants perceived the chest placement of the elastic band to provide a greater sense of security in terms of safety whilst performing the exercises compared to the hip placement. This perception may be related to the points addressed previously with the chest intervention being less challenging in terms of the balance required to perform the exercises compared to that with band placement at the hip. Sex was a factor associated with the perceived comfort of the exercise selection, with females expressing how the chest harness felt uncomfortable due to the placement of the elastic band high on the anatomical position of the chest, whereas this was not a concern for males. This suggests that the chest intervention may not be feasible, or at least less preferred, than the hip intervention for some females. Regardless, both locations of the elastic band placement (chest and hip) were reported to be challenging with regard to balance. Thus, the exercise equipment and the elastic band placement should be taken into consideration or be modified to suit individuals in future interventions.

#### 4.1.4. Perceptions of Intervention

The personal preferences reported for the exercise selection were associated with the ability to perform the exercises, with participants preferring to perform the exercises that were less difficult and at which they therefore felt more capable and confident to perform. This ease provided participants with a greater sense of enjoyment due to being able to perform the selected exercises [46]. The importance of enjoyment in PA is recognised as an essential aspect of engagement for older adults, which should be considered in PA interventions for older adults [46]. Additionally, enjoyment is highlighted in the SEM as a factor that makes activities accessible and appealing [7]. The PA interventions have the potential to provide a graded challenge to allow individuals to become proficient and efficacious in the exercises over time, which has previously been highlighted as a motivational factor, and should thus be considered for older adults [44].

#### 4.1.5. Perceptions of Exercise Intensity

The PA intervention was delivered at a low/moderate intensity, which can be altered to gradually increase the intensity over time to provide a continuously challenging program as the neuromuscular system adapts [47]. The adaptability of the exercises would allow participants to progress at their own pace with the activity, which has been highlighted as an important aspect in engaging older adults in PA within the SEM [7,48]. Including this aspect of adaptability in PA interventions for older adults enables individuals with varying needs to participate in PA and allows older adults to exercise within the limits of their own abilities [44].

#### 4.1.6. Exercise Intensity: Heart Rate (HR) and Perceived Exertion (RPE)

Heart rate and RPE measures indicated that the intensity of the PA intervention increased to a low-to-moderate level. Although moderate-to-vigorous levels of PA are recommended in the PA guidelines for older adults [16], the physical and mental health benefits of increasing sedentary levels of PA may be overlooked. The importance of providing older adults with the confidence to engage in PA to be more active is essential to promote behaviour changes and leads to increased PA levels over time [44,45]. The current PA interventions can be tailored for varying physical abilities and can be modified to increase the difficulty and therefore intensity of the exercises over time to meet individual needs.

#### 4.1.7. Reflection on Aspects of Socialisation

Within this study, all participants reported a positive experience from engaging in the PA. A contributing factor was the social interaction aspect, which emerged as a general theme (Section 3.5.2). The interaction with the researchers/supervisors was a facilitator of engagement in the intervention. The importance of the social element has previously been identified in the self-determination theory [10,11] as a facilitator that motivates behaviour, which may promote behaviour change in PA engagement [49]. A prominent theme was that the group setting/supervision was the preferred environment of participants to engage and perform the PA intervention, which is consistent with previous research indicating the importance of the social aspect in providing participants with motivation, enjoyment, and social interaction. Other studies have shown that theses aspects are important factors to be considered in PA interventions to make them appealing and engaging for older adults [50]. The SEM demonstrates the importance of this social element in activities as an essential motivator for older adults to adhere to PA [7]. Furthermore, as this research was conducted during COVID-19 restrictions, group contact may have been a concern. However, some of the participants wanted the group contact, which may explain the personal preference for a preferred group setting [27]. As older adults may be at high risk of social isolation [51], the opportunity for social engagement is an important aspect to be considered in PA interventions to influence relationships and improve social connectedness to provide a positive experience [52]. Future considerations of a group setting for the current PA interventions would be advantageous to make the intervention accessible for older adults [7,53].

#### 4.1.8. Organisation and Tailoring

The opinion about the exercise organisation in terms of the sets and repetitions varied for each participant. This was likely a result of each participants’ previous experience of PA [44]. This further enhances the importance of individual tailoring and meeting individual needs, which has previously been stated to be a safe and effective approach to improving physical outcomes in older adults [54,55]. The perceived suitability of the intervention was considered to ensure that the intervention was suitable for older adults. Participants revealed that they felt the intervention was suitable for themselves and others in their age group as they were aware of the deterioration of balance control in ageing. The participants in the older age group (65–84 years) reported perceived benefits of performing the exercises, associated with their own abilities to perform the exercises. However, participants in the younger age bracket (50–64 years) thought that the exercises would be beneficial at a later age when their balance control would be decreased, although at this age (50–64 years), this could possibly be considered as too late. Providing the opportunity for an early preventative PA intervention in middle-aged adults to improve and maintain balance performance may be key to delaying declines in daily functioning and falls later in life [56,57].

Importantly, the opinions and ideas of older adults have contributed to shaping the design of the intervention. This included ways in which the equipment can be altered to provide comfort and ways to progress the intervention to make it more challenging, such as incorporating weights, incorporating the exercises in a multicomponent intervention, and increasing the resistance of the elastic band to further challenge balance. The inclusion of progressively challenging balancing exercises is essential in such interventions as the body’s sensory systems are highly adaptive [58]. Modifications should be considered to suit individual preferences in PA interventions for older adults, for example, by altering the resistance of the elastic band and having participants stand out further from the anchor point to increase the resistance and instability and therefore make the exercises progressively more challenging to improve and adapt balance control strategies [59]. The practice of postural control strategies helps to improve and challenge balance, supporting the ideology of the PA intervention [35].

### 4.2. Strengths and Limitations

An important strength of the present study is that it provided rich information about the perceptions of the PA design of older adults and utilised the participants’ perceptions/feedback to shape the intervention. This is useful for directing future PA interventions for older adults to make them accessible, enjoyable, and appropriate. Further, the present study shares the process of how to undertake this research, involving older adults in the development of shaping and designing interventions to meet their needs, as called for in the literature [7]. This will be helpful for other stakeholders seeking to identify the preferences for the exercise section, safety, social, and enjoyment factors. These research findings will be disseminated to older adults and the providers of services for older adults through local PA networks in Derbyshire and the East Midlands (UK). Furthermore, whilst this study evaluated the perceptions of older adults, participants’ balance performances were not measured directly using a balance performance measure. This research was conducted during COVID-19 restrictions with safety precautions, although the recruitment to the study was limited to those individuals who felt confident in the university setting during this time.

## 5. Conclusions

In this study, the insights, opinions, and preferences regarding the novel **elastic** band resistance PA protocol provided valuable information for designing and shaping future PA interventions suitable for older adults. The importance of including security, safety, and the option of regressions and progressions in PA tailored to individual needs/abilities should be considered within such interventions. The enjoyment, socialisation, and perceived benefits of performing PA are important aspects and motivating factors in older adults’ decision to engage in PA. Given the outcomes, it would suggest that the PA intervention is feasible and appropriate for **older adults** to perform at a low-to-moderate exercise intensity. Ongoing efforts to involve and include older adults in the intervention design to meet the needs and preferences of older adults are of significance to enable PA interventions to be successful. This study contributes to the development of a novel PA intervention using elastic band resistance training to challenge balance and utilised the perspectives of **older adults** in shaping an accessible, appropriate, and acceptable intervention to meet their needs. Furthermore, the current intervention has the potential to improve balance in **older adults** and therefore prevent falls and mitigate fall occurrences, although further research is required to identify the effects on balance and postural sway patterns to confirm the efficacy of the program in mitigating common fall risk characteristics.

## Figures and Tables

**Table 1:** Descriptive characteristics of participant demographics.

Demographic Characteristics	All Participants (*n* = 23)
Age (years)	66.5 ± 8 (51–81 years)
Body mass (kg)	83 ± 15
Height (cm)	165 ± 12
IPAQ	High
MMSE	29 ± 1
IADL	8 ± 1
FES-I	9 ± 4
Mini BESTest	26 ± 3

*Notes: values presented as mean ± standard deviation. IPAQ: international physical activity questionnaire, FES-I: falls efficacy scale, IADL: International Activity of Daily Living questionnaire, MMSE: mini mental state examination, Mini BESTest: mini balance evaluation test.*

**Table 2:** Demographic profile of the participants.

Participant	Gender	Ethnicity	Age Group (Years)
1	M	White British	75–84
2	F	British	75–84
3	F	British	65–74
4	F	White British	75–84
5	M	British	65–74
6	F	White British	50–54
7	F	White British	65–74
8	F	White British	65–74
9	F	White British	65–74
10	F	White British	55–64
11	F	White British	55–64
12	M	African	55–64
13	F	White British	65–74
14	F	White British	65–74
15	F	White British	65–74
16	M	White British	55–64
17	F	White British	75–84
18	M	White British	50–54
19	M	White British	75–84
20	M	White British	55–64
21	M	White British	50–54
22	M	White British	65–74
23	M	White British	65–74

*Notes: gender M = male, F = female.*

**Table 3:** Comfort scores of the exercises performed at the hip region.

	Hip Region (%)					
Exercise	1	2	3	4	5	Mean ± SD Score
Forward step	0	4.3	4.3	13.4	78.0	4.7 ± 0.8
Forward tandem steps	8.6	8.6	13.0	17.4	52.4	4 ± 1.4
Forward tandem hold	8.6	4.3	17.4	4.3	65.4	4.1 ± 1.4
Side steps	0	0	17.4	21.7	60.9	4.4 ± 0.8
Upper body rotation	0	0	4.3	26.2	69.5	4.7 ± 0.6
Backward step	0	0	21.7	26.2	47.8	4.2 ± 0.9
Backward tandem steps	8.6	4.3	17.4	26.2	43.5	3.9 ± 1.3
Backward tandem hold	8.6	0	21.8	21.8	47.8	4 ± 1.2

*Notes: Score = percentage score out of 23 participants on a likert scale of 1–5, 1 being very uncomfortable and 5 being very comfortable. Average values presented as mean ± standard deviation.*

**Table 4:** Comfort scores of the exercises performed at the chest region.

	Chest Region (%)					
Exercise	1	2	3	4	5	Mean ± SD Score
Forward step	4.3	4.3	4.3	26.2	60.9	4.3 ± 1.1
Forward tandem steps	8.6	8.6	8.6	26.2	48	4 ± 1.3
Forward tandem hold	8.6	4.3	13.1	21.7	52.3	4 ± 1.3
Side steps	8.6	4.3	0	30.4	56.7	4.2 ± 1.2
Upper body rotation	4.3	4.3	0	26.2	65.2	4.4 ± 1
Backward step	4.3	4.3	8.6	26.2	56.6	4.3 ± 1.1
Backward tandem steps	13	8.6	4.3	26.2	46.9	3.9 ± 1.3
Backward tandem hold	13	4.3	8.6	21.7	52.4	4 ± 1.4

*Notes: Score = percentage score out of 23 participants on a likert scale of 1–5, 1 being very uncomfortable and 5 being very comfortable. Average values presented as mean ± standard deviation.*

**Table 5:** Enjoyment scores of the exercises performed at the hip region.

	Hip Region (%)					
Exercise	1	2	3	4	5	Mean ± SD Score
Forward step	0	4.3	4.3	17.4	74	4.6 ± 0.8
Forward tandem steps	13	0	13	13	61	4.1 ± 1.4
Forward tandem hold	13	0	8.6	13	65.4	4.2 ± 1.4
Side steps	0	4.3	4.3	34.8	56.6	4.4 ± 0.8
Upper body rotation	0	0	8.6	17.4	74	4.7 ± 0.6
Backward step	4.3	4.3	8.6	26.2	56.6	4.3 ± 1.1
Backward tandem steps	8.6	0	13	17.4	61	4.2 ± 1.2
Backward tandem hold	8.6	0	8.6	21.8	61	4.3 ± 1.2

*Notes: Score = percentage score out of 23 participants on a likert scale of 1–5, 1 being not enjoyable and 5 being very enjoyable. Average values presented as mean ± standard deviation.*

**Table 6:** Enjoyment scores of the exercises performed at the chest region.

	Chest Region (%)					
Exercise	1	2	3	4	5	Mean ± SD Score
Forward step	4.3	0	4.3	30.4	61	4.4 ± 0.9
Forward tandem steps	13	0	4.3	30.4	52.3	4.1 ± 1.3
Forward tandem hold	13	0	0	34.8	52.2	4.1 ± 1.3
Side steps	4.3	0	0	30.4	65.3	4.5 ± 0.9
Upper body rotation	4.3	4.3	0	21.7	69.7	4.5 ± 1
Backward step	4.3	0	0	30.4	65.2	4.5 ± 0.9
Backward tandem steps	13	0	4.3	26.1	56.6	4.1 ± 1.4
Backward tandem hold	13	0	0	30.4	56.6	4.2 ± 1.3

*Notes: Score = percentage score out of 23 participants on a likert scale of 1–5, 1 being not enjoyable and 5 being very enjoyable. Average values presented as mean ± standard deviation.*

**Table 7:** Difficulty scores of the exercises performed at the hip region.

	Hip Region (%)					
Exercise	1	2	3	4	5	Mean ± SD Score
Forward step	0	0	13	21.7	65.3	4.5 ± 0.7
Forward tandem steps	4.3	8.6	30.4	30.4	26.3	3.7 ± 1.1
Forward tandem hold	4.3	17.4	13	43.5	21.8	3.6 ± 1.2
Side steps	4.3	4.3	4.3	26.1	61	4.3 ± 1.1
Upper body rotation	0	4.3	8.6	7.4	69.7	4.5 ± 0.8
Backward step	0	8.6	8.6	26.1	56.7	4.3 ± 1
Backward tandem steps	8.6	4.3	21.7	43.5	21.9	3.7 ± 1.2
Backward tandem hold	8.6	8.6	13	43.5	26.3	3.7 ± 1.2

*Notes: Score = percentage score out of 23 participants on a likert scale of 1–5, 1 being extremely difficult and 5 being extremely easy to perform. Average values presented as mean ± standard deviation.*

**Table 8:** Enjoyment scores of the exercises performed at the hip region.

	Chest Region (%)					
Exercise	1	2	3	4	5	Mean ± SD Score
Forward step	4.3	0	0	26.1	69.6	4.6 ± 0.9
Forward tandem steps	4.3	8.6	17.4	30.4	39.3	3.9 ± 1.2
Forward tandem hold	4.3	8.6	17.4	30.4	39.3	3.9 ± 1.2
Side steps	4.3	4.3	4.3	34.8	52.3	4.3 ± 1.1
Upper body rotation	0	4.3	8.6	17.4	69.7	4.5 ± 0.8
Backward step	0	4.3	13	30.4	52.3	4.3 ± 0.9
Backward tandem steps	8.6	4.3	21.7	34.8	30.6	3.7 ± 1.2
Backward tandem hold	8.6	4.3	21.7	34.8	30.6	3.7 ± 1.2

*Notes: Score = percentage score out of 23 participants on a likert scale of 1–5, 1 being extremely difficult and 5 being extremely easy to perform. Average values presented as mean ± standard deviation.*

**Table 9:** Participants’ preferences for the location of the elastic band.

Preference	Elastic Band Placement (*n* = 23)		
To perform again Safety	**Chest**	**Hip**	**Either**
	11	7	5
	12	7	4

*Notes: chest: elastic band placed at the chest region. Hip: elastic band placed at the hip region and either: Participants did not have a preferred placement of the elastic band.*

## Data Availability

The data are not publicly available due to ethical restrictions. Please contact the corresponding author for further information.

## Supplementary Materials

The following supporting information can be downloaded at: https://www.mdpi.com/article/10.3390/ijerph191710907/s1, Supplementary File S1: Refinement of methods during piloting; Supplementary File S2: Quotations from interview schedules.
