# Supplementary material for: Feasibility, Psychosocial Effects, Influence, and Perception of Elastic Band Resistance Balance Training in Older Adults
Source: Int J Environ Res Public Health. 2022 Sep 1;19(17):10907. doi: 10.3390/ijerph191710907 (PMC9517780; doi:10.3390/ijerph191710907)
Supplement: Supplementary file 1 [file ijerph-19-10907-s001.zip › ijerph-1859010-supplementary.pdf]

Supplementary file S1: Refinement of methods during piloting

During the piloting phase of this study the following changes were made to the methodology and the equipment of the physical activity intervention.

Alterations to the Exercise Equipment:

- Exercise band originally anchored and attached at hip level to a squat rack. This was then altered to be attached and anchored at ground level to increase the difficulty of the intervention and further challenge balance.
- On the chest region the placement of the exercise band was altered to be placed above the chest region and underneath the armpit due to comfort.
- On the chest region the chest harness in which the exercise band attached was altered to add Velcro to attach the exercise band securely and to prevent from slipping during the intervention.
- On the hip and chest region the exercise equipment was altered to use a foam/seatbelt cover padding around the exercise band due to comfort and to avoid rubbing of the exercise band on the skin.
- Participants were originally required to lift the exercise band/equipment over the head this was altered for the participant to step into the equipment due to ease and comfort.

Alterations to the Physical Activity Intervention:

- Change of original exercises – sidestep exercise to have exercise band at the side (of the hip) instead of front due to comfort.
- Tandem walk exercise was replaced into a tandem hold exercise due to safety.
- All tandem exercises in the forward direction were altered to hold the arms out to the sides of the body when performing.
- During all exercises participants were required to alter the leading foot throughout each exercise.
- All exercises performed in the backward direction- Participants required to hold the harness at the top when performing exercises backwards due to safety of the equipment and discomfort.

Supplementary file S2: Quotations from interview schedules

Additional responses continued from the results of the interview schedules

3.3. Participants perceptions of the exercise selection

3.3.1. The PA intervention and balance

Participants reported their perceptions on the exercises that challenged balance whilst performing them using an elastic resistance band. This is illustrated by the following quotes:

- "I think they were because I wobbled a bit, didn't I? They were more difficult. I just couldn't feel that I had my balance as well" (backward exercises). (P10)
- "Both of them were really challenging (tandem hold front and back) because you're moving but then holding it for four seconds". (P11)

Participants reported how they perceived some of the exercises did not challenge their balance as such:

- "I think the twist (upper body rotations), even though you are moving around, I didn't find that quite so challenging and I thought the side-to-side ones (side steps) were pretty easy, but maybe because I'm used to them". (P11)
- "I think the side to side (side steps) were not a balance one". (P16)

Overall, the PA intervention was perceived to challenge balance and stability of most participants. This is demonstrated in the following quotes:

- "Yes, (it was challenging) because I was occasionally getting a wobble-on, as you probably noticed looking at the legs". (P23)
- "They all did challenge my balance". (P12)
- "Yes, yes they did, it's a nice kind of controlled way to find out if you've got any problems where they are". (P21)

3.3.2. Comfort of the exercise selection

- Lower scores on the tandem steps, tandem holds and forward step "…because I was just a bit wobbly on those". (P14)

Furthermore, holding the arms out to the sides during the exercises was an element to the exercises that participants felt was uncomfortable:

- "Holding my arms out, that hurt me like beyond just the weight to my arms. Just holding out for a period of time. I felt pain there like a lactic type build up in my arms. I wanted to let my arms drop and loosen them off and wiggle them". (P20)
- "The hardest for me was the one in front of the other feet (tandem steps and tandem holds) and sometimes holding your arms out like that – doing it makes your arms ache, but that's the problem people have to overcome, isn't it?". (P22)
- "I wouldn't say any of them were uncomfortable. That is probably from my point of view". (P23)

3.3.3. Preference of the exercise selection

The participants reported their preference on the exercise selection:

- "I would say upper body rotation and I would say the forward walk (forward step) and the forward tandem exercise. I think because it just challenges me to stay upright, focus and balance properly". (P3)

Several participants reported what they disliked about the exercises:

- "I didn't dislike any of them except for these two" (tandem steps and tandem holds). "I don't want anything to do with tandems, as long as it wasn't the tandem it was okay". (P9)
- "Certainly, a not favorite one would be the one where you got the feet in front of one in front of the other" (tandem steps and tandem holds). (P22)

- “If I had to put something at the bottom, it would probably be that one” (upper body rotation). “But that just me”. (P23)

3.3.4. Difficulty of the exercise selection

- Upper body rotation: “They're difficult but you feel they’re difficult to what you make them. They're not difficult to do, I wouldn't say, but they are difficult if you raised with the band. It's what you put into it that makes it difficult on them”. (P6)

In other cases, participants did not perceive the exercises to be difficult to perform:

- “It's easier to go forward than backwards”. (P7)

3.3.6. Organisation of the exercise selection

Participants reported on the way that the exercises were organised. The repetitions and sets of exercises were perceived to be adequate. Participant responses were as follows:

- “Just right. It was spot on. Everything was spot on”. (P13)

However, in some cases, participants felt that the repetitions and sets of exercises could have been increased:

- “Actually, I was thinking more of it will be good”. (P12)
- “Well, I wouldn't mind more but whether anybody else would or not I don't know. I mean, there's enough range there”. (P8)

3.3.7. Suitability of the PA intervention

- “Yeah, absolutely yeah, they need good balance”. (P5)

In some cases, participants felt the exercises were not age appropriate:

- “No, I’m just too old”. [for the exercises] (P2)

3.3.8. Perceived benefits of the PA intervention:

- “Well, with being large, I feel like it’s benefiting my waist and I feel it pulling in my waist and being beneficial for what I want it to do; toning up the muscles. It’s very good for toning”. (P6)
- “The one that I thought was sort of using different muscles that I might not use as much as I ought to were the side ones (side steps). I think, I don't know, I guess I, I'd say that I don’t know what the term is but feeling beneficial, you know?”. (P19)

3.3.9. The Psychosocial impact of the PA intervention

Participants reported if anxiousness was an aspect that they experienced when performing the exercises. Participant responses to the question “Did you feel anxious at all?” are quoted below:

- “Yes, a bit. There were a couple (of exercises) that I struggled to make my feet do what your feet had done. The word tense I think is good because you're not relaxed and you need to relax to listen and to absorb what's being said to you and I probably felt a bit tense, so I wasn't listening correctly. It is as though your brain and your muscles just won't do any more than that”. (P2)
- “No, I was very confident about doing the exercises and I was very confident that I got the support there from both of you”. (P3)

Participants reported if the exercises made them focus their attention on the task in hand:

- **“**It just requires brains as well as movement otherwise you’ll trip up”. (P13)

All the participants reported a positive experience in participation and that they would perform the exercises again:

- “Yeah, I would do it if I was in a gym and I've got a plan, I would do it because I liked it” (P11)
- “Yeah, actually I’ve been thinking of, I’m just going to incorporate that in my training program”. (P12)
- “Loved it, I just really enjoyed it. I’d definitely do them (exercises) against my mate, I would then we would have a competition”. (P16)
- “Thoroughly enjoyed it. Totally, lovely way to spend the lunch time, it’s been a lovely experience”. (P2)

3.4 Opinions of the exercise equipment

The participants reported their opinions on the safety aspect and what they liked about performing the exercises with the elastic band placed at the chest:

- “You can lean into the band as well and it just takes your weight, doesn’t it? It felt more secure leaning back on it”. (P6)
- “You felt a bit safer when it was up here”. (P14)

Furthermore, participants discussed what they disliked about performing the exercises with the elastic band placed at the hip:

- I found that where the pressure was around my lower back was, um, slightly uncomfortable. I wasn't injuring myself. I was worried about my lower back on that one. It could be uncomfortable and I think that if I did it and kept doing it [backward exercises] that I would end up with lower back problems (P11)

Participants also stated what they disliked about the chest:

- “It was a little bit uncomfortable across the chest and if the harness slipped a little bit”. (P10)

In some cases, female participants reported how they felt restricted wearing the chest harness equipment with the elastic band placed at the chest:

- “I felt as though it was crushing my bosoms [breasts] as well, which I didn't like but it may just be something to do with me. Perhaps a man wouldn't be bothered so much”. (P2)
- Was it uncomfortable? “I think probably, being female, yes. I think if you're male you wouldn't. I think that you wouldn't have the same sort of sensation”. (P4)
- “If I was male, it wouldn't feel that uncomfortable. It's that it feels as though you've got something pressing in all the time and it's just uncomfortable”. (P5)
- “It just restricts you when you've got a bust [breasts]”. (P6)
- “I know I've not got big, massive boobs [breasts] but I hate squashing what have got. So probably for a guy that might feel okay. I am conscious of, like, pressing on soft tissue. I think that's what it is”. (P11)

3.4.2 Difficulty of exercises performed with the elastic band placed at the hip.

The difficulty of performing the exercises was further discussed in terms of the elastic band placement:

Hip Region

- “With the hip it didn't fit – didn't feel as it was fitting, whether it's because I’m bigger? It just didn't feel as secure, not as comfortable”. (P8).

Chest Region

- “You just have to lean into either back or forward you know what I mean. I've got a good strong tummy”. (P20)

3.4.3. Preferred elastic band placement

- “I can see the benefits of both that's the thing. If I was going in for a quick workout and I haven't got time to do both I’d probably do the chest one. I think that I would get on that quick workout probably get feel like I've got a whole body instead of just the lower body”. (P11)

The safety element in terms of the exercise equipment was an aspect reported by participants with the elastic band placed at the chest providing a greater sense of safety compared to the chest.

- “I'm going to say neither because they were both the same for me”. (P22)
- “The chest harness felt safer than just the hip band I would that would be my only comment”. (P19)
- “Probably safer with the chest”. (P23)

3.5 Environment

3.5.1 Safety factors:

- “Yeah, fine, well, I've got two good trainers”. (P5)

- “I'm not worried about the environment were in i.e. this physical education type, it doesn’t bother me”. (P20)
- “I thought it was well explained and it was well done”. (P18)
- “I was very confident that I got the support there from both of you”. (P3)

3.5.2 Preferred setting

- “In a group setting definitely. I do group stuff and to be honest I'm not very good at self-motivating at home”. (P15)
- “Supervision or group setting. I can't motivate myself”. (P13)
